# Traumatic trigeminal neuropathy after whiplash injury: A case report

**DOI:** 10.1097/MD.0000000000029012

**Published:** 2022-03-11

**Authors:** Sung Ho Jang, Jeong Pyo Seo, Young Hyeon Kwon

**Affiliations:** a Department of Physical Medicine and Rehabilitation, College of Medicine, Yeungnam University, 317-1, Daemyungdong, Namku, Taegu, Republic of Korea; b Department of Physical Therapy, College of Health Sciences, Dankook University, Cheonan, Republic of Korea; c Department of Physical Medicine and Rehabilitation, College of Medicine, Yeungnam University, 317-1, Daemyungdong, Namku, Daegu, Republic of Korea.

**Keywords:** diffusion tensor tractography, head trauma, traumatic trigeminal neuropathy, trigeminal nerve, whiplash

## Abstract

**Rationale:**

Many studies using diffusion tensor tractography (DTT) have reported trigeminal neuropathy in various neurological diseases. However, no study on traumatic trigeminal neuropathy following whiplash has been reported.

**Patient concerns:**

A 51-year old female suffered an indirect head trauma resulting from a flexion-hyperextension injury. At approximately 30 minutes after onset, she began to sense a headache in the left frontal area and sensory changes in the left facial area, signs that intensified with the passage of time. At 7 days after onset, she visited the rehabilitation department of our university hospital and described the characteristics and severity of pain as follows: headache on the left frontal area including the forehead with intermittent squeezing and numbness sensations. Her visual analog scale pain score was 6 with her left cheek having a continuous, dull, swelling sensation (visual analog scale score: 1). On neurological examination, she revealed mild allodynia without hyperalgesia or somatosensory change on the head, cheek, tongue, and oral cavity.

**Diagnosis:**

Diffusion tensor imaging data were acquired 7 days after onset. On DTT, the left trigeminal nerve showed discontinuation in the middle portion compared to that of the right trigeminal nerve. Traumatic trigeminal neuropathy was diagnosed based on her clinical features and DTT findings.

**Intervention:**

She was prescribed carbamazepine (200 mg/day) and pregabalin (150 mg/day), and her facial pain was well-controlled to a tolerable level.

**Outcomes:**

These drugs were stopped after approximately 7 month's administration, however, she did not complain of facial pain.

**Lessons:**

By using DTT, we demonstrated traumatic trigeminal neuropathy in a patient with whiplash. We suggest that DTT would be a useful tool for detection of traumatic trigeminal neuropathy in patients who show clinical features of trigeminal neuropathy following whiplash.

## 1 Introduction

Whiplash is a bony and/or soft tissue injury resulting from acceleration–deceleration energy transfers in the neck.^[1]^ Recently, several studies have demonstrated brain injury following whiplash.^[2]^ Many studies have reported clinical evidence of facial symptoms which suggests the presence of trigeminal neuropathy after whiplash.^[3–9]^ However, because conventional brain magnetic resonance imaging (MRI) is limited in its capability to demonstrate trigeminal neuropathy, trigeminal neuropathy after whiplash could not be clearly diagnosed. By contrast, diffusion tensor tractography (DTT) which is a derivation of diffusion tensor imaging (DTI), allows 3-dimensional reconstruction and estimation of the trigeminal nerve.^[10]^ Many previous studies using DTT have demonstrated trigeminal neuropathy in various neurological diseases, particularly trigeminal neuralgia.^[11–20]^ However, no study on traumatic trigeminal neuropathy following whiplash has been reported.

In this study, we report on a whiplash patient who showed traumatic trigeminal neuropathy, which was demonstrated on DTT.

## 2 Case report

**Figure 1:**
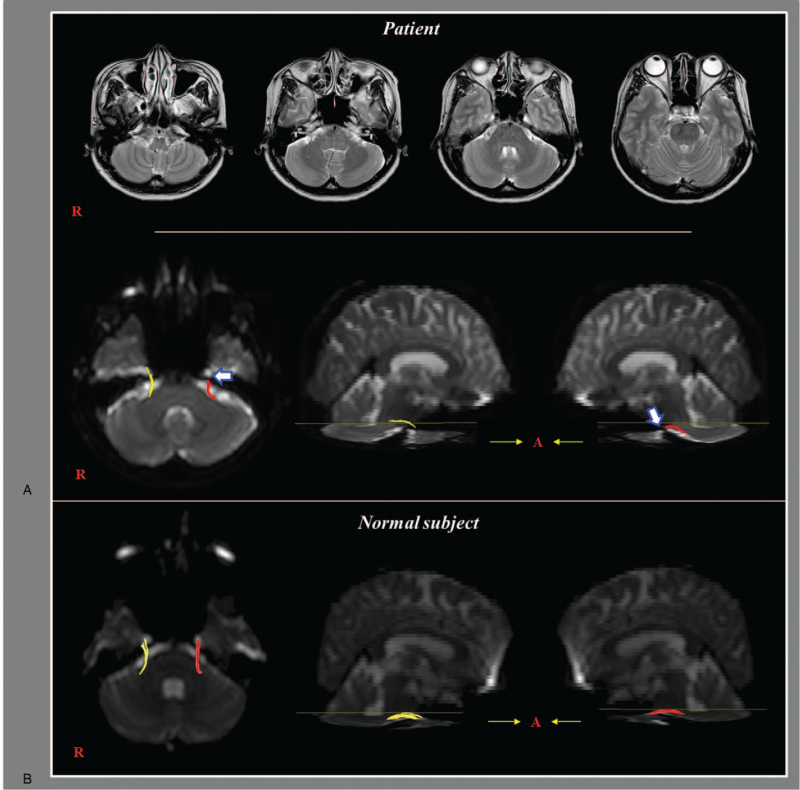
(A) T2-weighted brain magnetic resonance (MR) images obtained 7 days after whiplash onset show no abnormality (upper row). Results of diffusion tensor tractography: the left trigeminal nerve is discontinued (arrow) (lower row). (B) Results of diffusion tensor tractography for a control subject (53-year old female).

A 51-year old female with no history of neurological, physical, or psychiatric illness suffered an indirect head trauma resulting from a flexion-hyperextension injury after being hit from behind by a moving vehicle while stopping at an intersection. At the time of the head trauma, she did not experience loss of consciousness or posttraumatic amnesia, and her Glasgow Coma Scale was 15. A approximately 30 minutes after onset, she began to sense a headache in the left frontal area and experience sensory changes in the left facial area, signs that increased with the passage of time. At 7 days after onset, she visited the rehabilitation department of our university hospital, the characteristics and severity of her pain were as follows: headache in the left frontal area including the forehead; a spontaneous intermittent squeezing and numbness sensation (visual analog scale score: 6) with a spontaneous, continuous, dull, swelling sensation in the left cheek (visual analog scale score: 1). On neurological examination, she revealed mild allodynia without hyperalgesia or somatosensory change on the head, cheek, tongue, and oral cavity. In addition, she did not show weakness of the left masseter and temporalis muscles. Conventional brain MRI and DTI were recommended for the patient. Conventional brain MRI including T1-weighted, T2-weighted, and fluid-attenuated inversion recovery images, obtained at 7 days after onset, showed no abnormality (Fig. 1A). She was prescribed carbamazepine (200 mg/day) and pregabalin (150 mg/day), and her facial pain was well-controlled to a tolerable level. Her facial pain was gradually relieved with the passage of time. These drugs were stopped after approximately 7 month's administration, however, she did not complain of facial pain. The patient provided written and informed consent, and the study protocol was approved by the institutional review board of our university hospital.

The DTI data were acquired 7 days after onset by using a sensitivity-encoding head coil on a 1.5 T Philips Gyroscan Intera (Hoffman-LaRoche, Best, Netherlands) with single-shot echo-planar imaging and a navigator echo. For each of the 32 non-collinear diffusion-sensitizing gradients, 67 contiguous slices (acquisition matrix = 96 × 96; reconstructed to matrix = 192 × 192 matrix; field of view = 240 × 240 mm; repetition time = 10,398 ms; time echo = 72 ms; parallel imaging reduction factor (SENSE factor) = 2; echo planar imaging factor = 59; *b* = 1000 s/mm^2^; number of excitation = 1; and slice thickness = 2.5 mm) were acquired parallel to the anterior commissure-posterior commissure line. Eddy current-induced image distortions were removed by using affine multi-scale 2-dimensional registration as included in the Oxford Centre for the Functional Magnetic Resonance Imaging of the Brain software library (www.fmrib.ox.ac.uk/fsl).^[21]^ DTI-Studio software (CMRM, Johns Hopkins Medical Institute, Baltimore, MD) was used for evaluation of the trigeminal nerve. For nerve fiber delineation, the seed region of interest was placed on the prepontine cistern and the target region of interest was placed on isolated distal branches. Fiber tracking was performed by applying a fractional anisotropy threshold of >0.1 and a direction threshold <70°.^[10]^ On DTT, the left trigeminal nerve showed discontinuation in the middle portion compared to that of the right trigeminal nerve (Fig. 1).

## 3 Discussion

In this study, we detected a discontinuation of the left trigeminal nerve, an observation that was in accord with the trigeminal neuropathy symptoms exhibited by our patient with whiplash. The patient showed clinical features that satisfied the definitive criteria for presence of peripheral painful traumatic trigeminal neuropathy (i.e., A, spontaneous pain; B, develops within 3 months of an identifiable traumatic event; C, allodynia present; D, imaging or neurophysiology results demonstrate a neurologic lesion and its location; E, not attributed to other disorders). As a result, the traumatic trigeminal neuropathy signs appear to coincide with the DTT results showing a neuropathy of the left trigeminal nerve.^[22]^

Several studies have reported patients who presented facial pain after whiplash which appeared to be related to traumatic trigeminal neuropathy.^[3,5,6,8]^ In 1988, McGlone et al^[3]^ reported on a patient who showed delayed onset of facial pain after a whiplash. In 2011, Haggman-Henrikson et al^[5]^ found that 88% of whiplash patients complained of the face and jaw pain which were examined by questionnaire for pain. Subsequently, Genese^[6]^ reported on a patient who showed the right facial numbness and cheek pain after a whiplash. The patient's facial symptoms were alleviated by treating the right-sided strain of the trigeminal nerve. In 2019, Samim and Epstein^[9]^ reported on a patient who complained of shooting pain in the jaw, cheek, and forehead beginning 7 days after a whiplash. However, these previous studies could not demonstrate trigeminal neuropathy because the authors suspected trigeminal neuropathy based on questionnaire or neurological examination without radiologic evidences. Since introduction of DTI, many DTT-based studies have demonstrated trigeminal neuropathy in various neurological diseases including classical trigeminal neuralgia, brain tumor, neurovascular compression, and multiple sclerosis.^[11–20]^ As a result, to the best of our knowledge, this is the first case study to report on the presence of traumatic trigeminal neuropathy following whiplash. However, the limitation of this study regarding DTT should be considered; DTT can produce both false positive and false negative results throughout the white matter of the brain due to complex fiber configurations such as crossing or kissing fibers and/or partial volume effects.^[23]^

In conclusion, by using DTT, we demonstrated the presence of traumatic trigeminal neuropathy in a patient with whiplash. We suggest that DTT can be a useful tool for detection of traumatic trigeminal neuropathy in patients who show clinical features of trigeminal neuropathy following a whiplash.

## Author contributions

Sung Ho Jang, study concept and design, manuscript development, writing, data acquisition, and drafting/revising the image.

Jeong Pyo Seo, study support, data acquisition, and drafting/revising the image

Yougn Hyeon Kwon, critical revision of manuscript for intellectual content, drafting/revising the image, writing, and funding.

**Conceptualization:** Sung Ho Jang, Younghyeon Kwon.

**Data curation:** Jeong Pyo Seo, Younghyeon Kwon.

**Methodology:** Jeong Pyo Seo.

**Visualization:** Jeong Pyo Seo.

**Writing – original draft:** Sung Ho Jang.

**Writing – review & editing:** Younghyeon Kwon.

## References

[1] Spitzer WO Skovron ML Salmi LR. Scientific monograph of the Quebec Task Force on whiplash-associated disorders: redefining “whiplash” and its management. Spine (Phila Pa 1976) 1995;20:1S–73S. 7604354

[2] Jang SH Kwon YH. A review of traumatic axonal injury following whiplash injury as demonstrated by diffusion tensor tractography. Front Neurol 2018;9:57. 29472891 10.3389/fneur.2018.00057 PMC5809420

[3] McGlone R Morton RJ Sloan JP. Trigeminal pain due to whiplash injury. Injury 1988;19:366. 3255722 10.1016/0020-1383(88)90118-0

[4] Sterner Y Toolanen G Knibestol M Gerdle B Hildingsson C. Prospective study of trigeminal sensibility after whiplash trauma. J Spinal Disord 2001;14:479–86. 11723396 10.1097/00002517-200112000-00003

[5] Haggman-Henrikson B Gronqvist J Eriksson PO. Frequent jaw-face pain in chronic whiplash-associated disorders. Swed Dent J 2011;35:123–31. 22135943

[6] Genese JS. Osteopathic manipulative treatment for facial numbness and pain after whiplash injury. J Am Osteopath Assoc 2013;113:564–7. 23843380 10.7556/jaoa.2013.008

[7] Haggman-Henrikson B Lampa E Nordh E. Altered thermal sensitivity in facial skin in chronic whiplash-associated disorders. Int J Oral Sci 2013;5:150–4. 23867844 10.1038/ijos.2013.42 PMC3967328

[8] Peterson S. Differential diagnosis and treatment of bilateral facial pain after whiplash: a case report. Physiother Theory Pract 2015;31:442–9. 25671352 10.3109/09593985.2015.1010244

[9] Samim F Epstein JB. Orofacial neuralgia following whiplash-associated trauma: case reports and literature review. SN Compr Clin Med 2019;1:627–32.

[10] Kabasawa H Masutani Y Aoki S. 3T propeller diffusion tensor fiber tractography: a feasibility study for cranial nerve fiber tracking. Radiat Med 2007;25:462–6. 18026904 10.1007/s11604-007-0169-8

[11] Hodaie M Chen DQ Quan J Laperriere N. Tractography delineates microstructural changes in the trigeminal nerve after focal radiosurgery for trigeminal neuralgia. PLoS One 2012;7:e32745. 22412918 10.1371/journal.pone.0032745 PMC3295766

[12] Liu Y Li J Butzkueven H. Microstructural abnormalities in the trigeminal nerves of patients with trigeminal neuralgia revealed by multiple diffusion metrics. Eur J Radiol 2013;82:783–6. 23265178 10.1016/j.ejrad.2012.11.027

[13] Lummel N Mehrkens JH Linn J. Diffusion tensor imaging of the trigeminal nerve in patients with trigeminal neuralgia due to multiple sclerosis. Neuroradiology 2015;57:259–67. 25404413 10.1007/s00234-014-1463-7

[14] Lee CC Chong ST Chen CJ. The timing of stereotactic radiosurgery for medically refractory trigeminal neuralgia: the evidence from diffusion tractography images. Acta Neurochir (Wien) 2018;160:977–86. 29397449 10.1007/s00701-017-3449-9

[15] Moon HC You ST Baek HM. 7.0 tesla MRI tractography in patients with trigeminal neuralgia. Magn Reson Imaging 2018;54:265–70. 29305127 10.1016/j.mri.2017.12.033

[16] Lee YJ Moon HC Tak S Cheong C Park YS. Atrophic changes and diffusion abnormalities of affected trigeminal nerves in trigeminal neuralgia using 7-T MRI. Stereotact Funct Neurosurg 2019;97:169–75. 31537003 10.1159/000502222

[17] Chai W You C Zhang W. Diffusion tensor imaging of microstructural alterations in the trigeminal nerve due to neurovascular contact/compression. Acta Neurochir 2019;161:01. 10.1007/s00701-019-03851-2 31065894

[18] Chen ST Yang JT Weng HH Wang HL Yeh MY Tsai YH. Diffusion tensor imaging for assessment of microstructural changes associate with treatment outcome at one-year after radiofrequency rhizotomy in trigeminal neuralgia. BMC Neurol 2019;19:62. 30979362 10.1186/s12883-019-1295-5 PMC6460667

[19] Leal PRL Roch J Hermier M Berthezene Y Sindou M. Diffusion tensor imaging abnormalities of the trigeminal nerve root in patients with classical trigeminal neuralgia: a pre- and postoperative comparative study 4 years after microvascular decompression. Acta Neurochir (Wien) 2019;161:1415–25. 31049710 10.1007/s00701-019-03913-5

[20] Wu M Qiu J Jiang X. Diffusion tensor imaging reveals microstructural alteration of the trigeminal nerve root in classical trigeminal neuralgia without neurovascular compression and correlation with outcome after internal neurolysis. Magn Reson Imaging 2020;71:37–44. 32439427 10.1016/j.mri.2020.05.006

[21] Smith SM Jenkinson M Woolrich MW. Advances in functional and structural MR image analysis and implementation as FSL. Neuroimage 2004;23: (Suppl 1): S208–19. 15501092 10.1016/j.neuroimage.2004.07.051

[22] Benoliel R Zadik Y Eliav E Sharav Y. Peripheral painful traumatic trigeminal neuropathy: clinical features in 91 cases and proposal of novel diagnostic criteria. J Orofac Pain 2012;26:49–58. 22292140

[23] Yamada K Sakai K Akazawa K Yuen S Nishimura T. MR tractography: a review of its clinical applications. Magn Reson Med Sci 2009;8:165–74. 20035125 10.2463/mrms.8.165

